# Patient acceptance of teleneurology across neurologic conditions

**DOI:** 10.1007/s00415-024-12200-y

**Published:** 2024-02-23

**Authors:** Courtney R. Seigel, Holly Martin, Grace Bastin, Laura J. Myers, Stan Taylor, Francis Pike, Jayne Wilkinson, Linda S. Williams

**Affiliations:** 1 Indiana University School of Medicine, Indianapolis, IN USA; 2 Richard L. Roudebush VAMC HSR&D EXTEND QUERI, Indianapolis, IN USA; 3 Regenstrief Institute, Inc., Indianapolis, IN USA; 4 Department of Biostatistics, Indiana University School of Medicine, Indianapolis, IN USA; 5 Corporal Michael J Crescenz VAMC, Philadelphia, PA USA; 6 Department of Neurology, University of Pennsylvania, Philadelphia, PA USA; 7 Department of Neurology, Indiana University School of Medicine, Indianapolis, IN USA

**Keywords:** Teleneurology, Telemedicine, Patient satisfaction, Comorbidity, Neurology

## Abstract

**Introduction:**

Patient acceptability with outpatient teleneurology has been reported within specific conditions, but less is known about acceptability across neurologic conditions. The study objective was to compare the acceptability of teleneurology between patients with various neurological conditions and determine what other factors influence acceptability.

**Methods:**

This was a prospective study of Veterans who completed new outpatient teleneurology visits with the Department of Veterans Affairs National Teleneurology Program. Visits were conducted via video to home or video to the outpatient clinic. Patient acceptability was assessed via telephone interview two weeks post-visit. Acceptability was a summed score (3–21) of three 7-point Likert questions (higher = more acceptable). Clinical diagnosis categories were based on the neurologists’ ICD10 diagnosis code. Acceptability score was modeled using a censored Tobit model controlling for demographics, type of tele-visit, medical comorbidity, and ICD10 category.

**Results:**

In FY 2021, 277 of 637 (43.5%) patients completed an interview with analyzable acceptability data. Of these 277, 70 (25.3%) had codes indicating headache, 46 (16.6%) movement disorder, 45 (16.2%) general symptoms, and 116 (41.9%) for all other categories. Mean patient acceptability was 18.3 (SD 3.2). There was no significant difference in scores between these groups. The only factor independently related to acceptability was medical comorbidity, with higher comorbidity associated with higher acceptability scores.

**Discussion:**

Patients find their outpatient teleneurology experience highly acceptable independent of neurologic condition. Those with more comorbidity report higher acceptability. Use of teleneurology may be useful and acceptable across many outpatient neurologic conditions including for more medically complex patients.

**Supplementary Information:**

The online version contains supplementary material available at 10.1007/s00415-024-12200-y.

## Introduction

Worldwide there is a shortage of neurologists [1]. In the US, this shortage is exacerbated by a rapidly aging population [1]. Neurologists are often also concentrated in urban areas, leaving patients in rural locations without easy access to a neurologist [2]. A recent analysis of Medicare data has shown that while the availability of neurologists varies widely across geographic regions, the prevalence of neurologic conditions does not [2]. This combination of a neurologist shortage, an aging population, and a large rural population in the US results in many patients unable to access the neurological care they need. The use of telemedicine to provide neurological services may help bridge these gaps in care. For these reasons, in 2019 the Department of Veterans Health Administration’s Office of Rural Health developed the National Teleneurology Program (NTNP) to provide access to specialty neurology care for Veterans in rural and under-resourced areas of the country. Implementation of this program has led to more timely neurologic care and a decrease in the growth of non-VA community care neurology consults [3].

The origins of teleneurology (TN) lie in the use of telemedicine to provide acute stroke care with the term telestroke introduced in 1999 [4]. Since that time, the use of telemedicine to provide other neurologic services has expanded, but less is known about the provision of telemedicine for other neurologic conditions than for telestroke care [5]. With limited options for in-person care in the early phases of the COVID-19 pandemic, utilization of TN increased greatly for outpatient care and for conditions other than stroke such as headache [7, 8]. TN use is likely here to stay despite difficulties that remain in payment models and care delivered across state lines [9]. The safety and efficacy of TN have been repeatedly demonstrated for stroke care and literature is beginning to emerge regarding other neurological conditions [5]. Prior research suggests that TN is safe and effective for many neurological conditions such as headache, movement disorders, and seizures [5, 10–12]. As a whole, it has also been shown to have many advantages such as increased access to care, reduced cost for the patient, decreased wait times, and reduced burden on the provider [5, 6].

Much of the existing TN literature focuses on the application of TN in patients with a specific condition. It is conceivable that some conditions or situations are better suited to TN than others, but as telehealth use expands, it may be helpful to understand the experiences of patients with a wider variety of neurologic conditions. To our knowledge, only one study has been conducted that compares patient satisfaction with TN across multiple neurologic conditions. This 2022 study of patients in New Delhi, India found that most patients were satisfied with their TN care during the COVID-19 lockdown, but that rates of satisfaction were highest amongst patients with stroke or a neuroimmunological condition [13]. Whether similar results would be observed in a US population of general neurology patients is unknown. The purpose of our analysis was to examine patient acceptability among general neurology patients referred to the VA NTNP and evaluate whether other patient and system factors also influence patient acceptability with TN care.

## Methods

### VA National Teleneurology Program

The NTNP program was funded by the VHA Office of Rural Health in October 2019. It includes 17 VA medical centers across the US with sites joining the program at different times; the first Veterans were seen by the program in October of 2020 (FY2021). Neurologists in the program are located across the country, not in a single physical “hub,” with a virtual administrative hub in Philadelphia. NTNP is a general neurology outpatient program that accepts referrals for clinical evaluation of any neurologic symptoms or conditions. Veterans may choose to have their TN visit through the NTNP in two different ways: video to home (VA Video Connect, VVC) and video in an outpatient clinic (Clinical Video Telehealth, CVT), which is typically conducted in a rural community-based outpatient VHA clinic setting with a dedicated space to conduct telehealth visits. In this analysis, we included data from all sites that joined the NTNP program before 2023.

### Patient selection

Veterans who completed a TN consult in the first six months of NTNP activity at their site were eligible for a patient satisfaction interview. All Veterans in the first three months of program implementation were contacted for a telephone interview, and a random 50% of Veterans in months 4–6 were contacted. Three call attempts were made within two weeks of the completed consult. If the Veteran was unable to complete the interview, a proxy who was also present during the telehealth visit could complete the interview.

### Patient survey

Telephone interviews included questions about satisfaction, similarity of the visit to an in-person visit, and likelihood of recommending a TN visit. These questions were a priori identified as the primary acceptability questions, based on our use of these questions in previous evaluations of the VA TeleStroke program. These questions were individually answered using a 7-point Likert scale (higher score indicating greater acceptability). The survey included other questions taken from VHA telehealth and other telehealth surveys regarding prior telemedicine experiences, technological difficulties, communication with the neurologist providing care, and the ways in which their TN experience did or did not improve access to care. Survey questions can be seen in supplementary materials Table 1.

**Table 1:** Characteristics of patients contacted to complete survey after their first NTNP consult

	Responded to interview			
	No (*N* = 338)	Yes (*N* = 299)	Total (*N* = 637)	*p*-value
Age				0.99^a^
*N*	338	299	637	
Mean (SD)	59.9 (16.5)	59.9 (16.1)	59.9 (16.3)	
Median	63	63	63	
Range	23.0, 93.0	25.0, 91.0	23.0, 93.0	
Sex, *n* (%)				0.91^b^
F	44 (13.0%)	38 (12.7%)	82 (12.9%)	
M	294 (87.0%)	261 (87.3%)	555 (87.1%)	
Race, *n* (%)				0.76^b^
Black	11 (3.3%)	14 (4.7%)	25 (3.9%)	
Other	4 (1.2%)	3 (1.0%)	7 (1.1%)	
Unknown	18 (5.3%)	13 (4.3%)	31 (4.9%)	
White	305 (90.2%)	269 (90.0%)	574 (90.1%)	
Rurality, *n* (%)				0.55^b^
Rural	173 (51.2%)	146 (48.8%)	319 (50.1%)	
Urban	165 (48.8%)	153 (51.2%)	318 (49.9%)	
Charlson Score				0.95^a^
*N*	338	299	637	
Mean (SD)	1.4 (1.93)	1.5 (2.11)	1.5 (2.02)	
Median	1	1	1	
Range	0.0, 10.0	0.0, 14.0	0.0, 14.0	
ICD10 Category, *n* (%)				
Headache	91 (26.9%)	71 (23.7%)	162 (25.4%)	
Movement Disorders	53 (15.7%)	49 (16.4%)	102 (16.0%)	
Symptoms	51 (15.1%)	48 (16.1%)	99 (15.5%)	
Neuropathy/Radiculopathy	30 (8.9%)	29 (9.7%)	59 (9.3%)	
Dementia	27 (8.0%)	15 (5.0%)	42 (6.6%)	
Other	19 (5.6%)	21 (7.0%)	40 (6.3%)	
Stroke/TIA	19 (5.6%)	18 (6.0%)	37 (5.8%)	
Seizures/Epilepsy	17 (5.0%)	19 (6.4%)	36 (5.7%)	
Pain	10 (3.0%)	9 (3.0%)	19 (3.0%)	
Multiple Sclerosis	7 (2.1%)	5 (1.7%)	12 (1.9%)	
Sleep Disorder	4 (1.2%)	5 (1.7%)	9 (1.4%)	
Abnormal Scan	4 (1.2%)	4 (1.3%)	8 (1.3%)	
Traumatic Brain Injury	3 (0.9%)	3 (1.0%)	6 (0.9%)	
Neuromuscular Disease	3 (0.9%)	1 (0.3%)	4 (0.6%)	
Tumors	0 (0.0%)	2 (0.7%)	2 (0.3%)	
Type of consult, *n* (%)				0.10^b^
VVC	156 (46.2%)	148 (49.5%)	304 (47.7%)	
CVT	180 (53.3%)	144 (48.2%)	324 (50.9%)	
Phone	2 (0.6%)	7 (2.3%)	9 (1.4%)	

*VVC* VA video connect, *CVT* Clinical Video Telehealth

^a^Kruskal-Wallis *p*-value

^b^Chi-Square *p*-value

### Outcomes and covariates

Demographic data and medical comorbidities were obtained using administrative data from the VA Corporate Data Warehouse (CDW). Race and ethnicity were identified using standard VA designations as part of the central warehouse data. The four categories for race were Black, White, Other, and Unknown. The Other categories included Asian, American Indian/Alaska Native, Native Hawaiian. The Charlson Comorbidity index (CCI) was calculated based on ICD10 diagnosis codes in the 1-year period prior to the TN consult [14]. Veterans were determined to live in rural or urban areas based on zip code at the time of consult request and using the standard VHA designation of rural or highly rural and urban. Neurological diagnosis was determined using the primary diagnosis code documented during the initial TN consult. We grouped diagnosis codes into common clinical groupings, for example, all types of headaches were included in the “Headache” category, and conditions like Parkinson’s Disease, essential tremor, and restless legs syndrome were included in the “Movement Disorders” category. Diagnosis codes indicating a symptom rather than a specific neurologic diagnosis (e.g., “dizziness and giddiness,” or “weakness”) were grouped into a “Symptoms” category. Patients in all other clinical diagnosis categories were combined into a miscellaneous category which was used as a reference group for this analysis. Type of telehealth modality (VVC or CVT) used for the visit was also recorded. For group comparison, the nine visits completed solely by telephone were combined with CVT visits. The primary outcome of interest was the Veteran acceptability of TN care, defined as the sum of the three primary acceptability questions with a total score ranging from 3 to 21 with higher numbers representing increased acceptability.

### Statistical analysis

The distribution of clinical characteristics across those who completed the survey or those who did not were tabulated and assessed using appropriate two sample tests for continuous and categorical variables respectively. We found the correlation between the three acceptability questions to be only modest (0.39–0.58) suggesting that they reflected unique aspects of acceptability and thus were appropriate for a summed score. Exact and non-parametric alternatives were utilized when sample size or the normality assumption appeared tenuous. Clinical characteristics and patient acceptability scores were tabulated across ICD10 groups and assessed as above after including only those patients who responded to all three acceptability questions. Since patient-reported satisfaction scores tend to exhibit a ceiling effect, we tested the effect of ICD10 groups on patient acceptability scores using a multivariable censored Tobit model. Although modeling included only patients who responded to all three acceptability questions, the tables also include participants who answered some, but not all three acceptability questions. All analyses were performed using SAS 9.4.

## Results

**Fig. 1:**
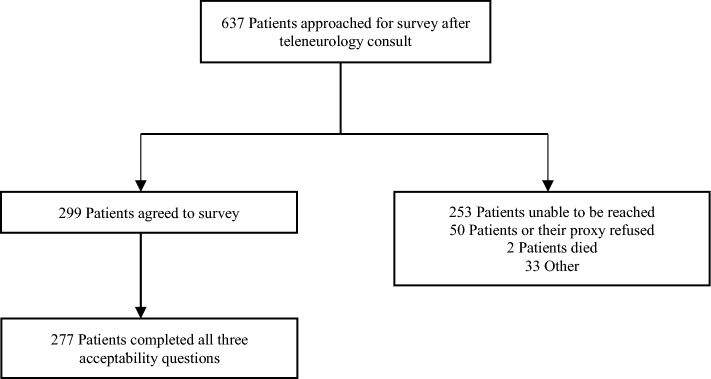
CONSORT diagram

Of the 637 patients contacted after their initial TN consult, 299 agreed to the interview (46.9% of those contacted) and 277 completed the three patient acceptability questions (43.5% of those contacted). Of the 637 patients contacted, 253 patients were unable to be reached, 50 patients or their proxy’s refused, 2 patients had died, and 33 did not participate for other reasons, such as incorrect phone number (Fig. 1).

Basic demographic data of patients who completed the survey are compared to the total patient group in Table 1. Overall, those who completed the acceptability interview were representative of the total group with no notable differences. Most patients (90.0%) in both groups were White males, indicative of the rural US Veteran population. The top three diagnosis groups were headache, movement disorder, and symptoms for both the total group and the acceptability group.

Of the 277 patients who completed the interview and had analyzable acceptability data, 70 (25.3%) were seen for headache, 46 (16.6%) for movement disorders, 45 (16.2%) for symptoms, and 116 (41.9%) for all other categories (see Table 1 for all ICD10 categories combined as “miscellaneous”). Comparison of demographic characteristics between these groups (Table 2) shows that the headache group was younger, had a higher proportion of females, had lower medical comorbidity, and were more likely to have a VVC visit than the other groups. Mean total patient acceptability score was 18.3 (SD 3.2) and there was no significant difference in acceptability between diagnosis groups, either in total score or in individual scores on the three acceptability questions (see Table 3). The type of visit, VVC or CVT, was not related to patient acceptability either (95% CI 0.5, – 0.6 to 1.6; *p* = 0.4). Table 4 includes descriptive data for additional survey questions demonstrating generally high ratings of the technical components of the visit, interactions with the neurologist, and positive assessment of the impact of telehealth on access to care. In the model of acceptability (Table 5), the only factor independently related to the acceptability score was medical comorbidity (95% CI 0.04, 0.04–0.07; *p* = 0.03), with higher comorbidity associated with higher acceptability scores.

**Table 2:** Characteristics of interviewed patients by ICD10 category

	Headache (*N* = 71)	Miscellaneous (*N* = 131)	Movement disorders (*N* = 49)	Symptoms (*N* = 48)	Total (*N* = 299)	*p*-value
Age						< 0.01^a^
N	71	131	49	48	299	
Mean (SD)	47.0 (14.1)	62.2 (15.0)	68.7 (11.4)	63.9 (14.8)	59.9 (16.1)	
Median	43	64	72	66	63	
Range	25.0, 79.0	26.0, 91.0	41.0, 89.0	25.0, 90.0	25.0, 91.0	
Sex, *n* (%)						< 0.01^b^
F	18 (25.4%)	16 (12.2%)	0 (0.0%)	4 (8.3%)	38 (12.7%)	
M	53 (74.6%)	115 (87.8%)	49 (100.0%)	44 (91.7%)	261 (87.3%)	
Race, *n* (%)						0.15^b^
Black	7 (9.9%)	7 (5.3%)	0 (0.0%)	0 (0.0%)	14 (4.7%)	
Other	4 (5.6%)	7 (5.3%)	2 (4.1%)	3 (6.3%)	16 (5.4%)	
White	60 (84.5%)	117 (89.3%)	47 (95.9%)	45 (93.8%)	269 (90.0%)	
Rurality, *n* (%)						0.14^b^
Rural	31 (43.7%)	59 (45.0%)	26 (53.1%)	30 (62.5%)	146 (48.8%)	
Urban	40 (56.3%)	72 (55.0%)	23 (46.9%)	18 (37.5%)	153 (51.2%)	
Weighted Charlson Score						< 0.01^a^
*N*	71	131	49	48	299	
Mean (SD)	0.5 (1.1)	1.9 (2.3)	1.9 (2.0)	1.7 (2.3)	1.5 (2.1)	
Median	0	1	1	1	1	
Range	0.0, 6.0	0.0, 14.0	0.0, 8.0	0.0, 8.0	0.0, 14.0	
Type of visit, *n* (%)						< 0.01^b^
CVT/Phone	25 (35.2%)	63 (48.1%)	33 (67.3%)	30 (62.5%)	151 (50.5%)	
VVC	46 (64.8%)	68 (51.9%)	16 (32.7%)	18 (37.5%)	148 (49.5%)	
Availability of Local Neurology, *n* (%)						0.21^b^
No	40 (56.3%)	80 (61.1%)	22 (44.9%)	24 (50.0%)	166 (55.5%)	
Yes	31 (43.7%)	51 (38.9%)	27 (55.1%)	24 (50.0%)	133 (44.5%)	

*VVC* VA video connect, *CVT* Clinical Video Telehealth

^a^Kruskal-Wallis *p*-value

^b^Chi-Square *p*-value

**Table 3:** Acceptability for interviewed patients by ICD10 category

	Headache (*N* = 71)	Miscellaneous (*N* = 131)	Movement disorders (*N* = 49)	Symptoms (*N* = 48)	Total (*N* = 299)	*p*-value
Overall, how satisfied were you with your Teleneurology visit?						0.8^a^
*N*	71	128	48	46	293	
Mean (SD)	6.3 (1.4)	6.3 (1.1)	6.2 (1.1)	6.2 (1.3)	6.3 (1.2)	
Median	7	7	7	7	7	
Range	1.0, 7.0	1.0, 7.0	2.0, 7.0	1.0, 7.0	1.0, 7.0	
To what extent was this Teleneurology consult like a face-to-face meeting?						0.1^a^
*N*	70	117	46	45	278	
Mean (SD)	5.5 (1.6)	5.8 (1.4)	6.0 (1.2)	5.4 (1.5)	5.7 (1.4)	
Median	6	6	6	6	6	
Range	1.0, 7.0	1.0, 7.0	2.0, 7.0	1.0, 7.0	1.0, 7.0	
Would you recommend Teleneurology to other Veterans like yourself?						1.0^a^
*N*	71	123	48	47	289	
Mean (SD)	6.3 (1.4)	6.4 (1.2)	6.4 (1.3)	6.2 (1.5)	6.3 (1.3)	
Median	7	7	7	7	7	
Range	1.0, 7.0	1.0, 7.0	1.0, 7.0	1.0, 7.0	1.0, 7.0	
Total acceptability score						0.6^a^
*N*	70	116	46	45	277	
Mean (SD)	18.1 (3.4)	18.5 (3.0)	18.7 (2.8)	17.7 (3.7)	18.3 (3.2)	
Median	19	19	19	19	19	
Range	3.0, 21.0	6.0, 21.0	9.0, 21.0	6.0, 21.0	3.0, 21.0	

^a^Kruskal-Wallis *p*-value

**Table 4:** Descriptive data from additional survey questions. These questions were scored from 1 to 5 with higher scores indicating more agreement with the statement (see supplemental files Table 1)

Additional Survey Question	Headache	Miscellaneous	Movement disorders	Symptoms
I was able to see the provider clearly by video				
*N*	71	117	44	44
Mean (SD)	4.9 (0.3)	4.8 (0.5)	4.8 (0.8)	4.8 (0.8)
I was able to hear the provider clearly by video				
*N*	71	116	44	44
Mean (SD)	4.7 (0.7)	4.7 (0.8)	4.6 (0.9)	4.6 (0.9)
I was able to ask questions directed to the Neurologist				
*N*	72	119	46	45
Mean (SD)	4.9 (0.3)	4.9 (0.5)	4.8 (0.6)	4.8 (0.7)
My provider explained things to me in a way that was easy to understand				
*N*	71	118	45	45
Mean (SD)	4.8 (0.5)	4.8 (0.6)	4.7 (0.5)	4.8 (0.7)
My provider listened to me during the appointment in a caring manner				
*N*	71	117	45	45
Mean (SD)	4.8 (0.5)	4.8 (0.5)	4.9 (0.3)	4.9 (0.6)
In general, telehealth reduces the need to travel long distances in order to meet with my provider				
*N*	70	112	45	45
Mean (SD)	4.6 (0.9)	4.5 (1.0)	4.6 (0.9)	4.5 (1.0)
In general, video visits help me get care that I couldn't access otherwise				
*N*	69	112	45	43
Mean (SD)	4.1 (1.3)	4.5 (1.0)	4.6 (0.8)	4.2 (1.3)

**Table 5:** Multivariate censored regression model of acceptability score

Variables	Estimate (95% CI)	*p*-value
ICD10 Category		0.53^c^
Headache	– 0.2 (– 1.6, 1.2)	
Movement Disorders	0.5(– 1.1, 2.0)	
Symptoms	– 0.9 (– 2.4, 0.6)	
Miscellaneous	Reference	
Age	– 0.01(– 0.05, 0.03)	0.68^c^
Sex		
Female	0.2 (– 1.4, 1.8)	0.83^c^
Male	Reference	
Weighted Charlson Score	0.35 (0.04, 0.65)	0.03^c^
VVC	Reference	
CVT/Phone	0.5 (– 0.6, 1.6)	0.36^c^
VVC	Reference	

*VVC* VA video connect, *CVT* Clinical Video Telehealth

^c^Adjusted *p*-value from a multivariable censored regression model to account for ceiling and confounding effects

## Discussion

Our study demonstrated that patients found their TN experience highly acceptable regardless of their neurologic condition and that the ICD10 diagnosis category was not significantly associated with the acceptability of TN care (see supplemental file for acceptability question descriptive data for additional ICD10 categories). However, medical comorbidity was independently related to acceptability with higher comorbidity associated with higher acceptability scores. This suggests that patients with more chronic medical conditions were more likely to find TN more acceptable. These findings suggest that TN is useful and acceptable to patients with a variety of neurologic conditions and that more medically complex patients may find it even more acceptable. As was demonstrated in an Italian study, utilization of telemedicine in the care of complex patients with chronic conditions has the potential to bridge gaps between hospital- and home-based care [15]. Given that many neurologic conditions are associated with mobility and cognitive issues, it is important to understand the utility of and patient experience with TN when these difficulties are present. We also found no relationship between the type of virtual visit and acceptability suggesting that a variety of TN delivery methods may be useful including virtual visits at home or in a medical office closer to the patient. Although the physician perspective was not the focus of this study, NTNP-referring physicians are also highly satisfied with TN care for their patients (data not shown) [3].

One of the biggest benefits of TN is its potential to increase patient access to neurologic care. Overall, there is a mismatch between the geographic location of neurologists and patients with neurologic conditions [2]. For certain conditions like multiple sclerosis, a high proportion of patients have access to a neurologist regardless of their location, but for other conditions, like dementia, access is lower in regions with lower neurologist density [2]. This issue of access is especially relevant for rural communities where there may be no local neurologist and the closest academic center may be hours away. Over 50% of the patients who completed the survey in this study did not have access to any local neurologist within the VHA system (Table 2) and 49% of them were classified as rurally residing.

Headache is among the most prevalent chronic neurologic conditions. Compared to many neurologic conditions, headache diagnosis and treatment are less dependent on ongoing physical exams. Thus, telemedicine may be especially well-suited for the evaluation and management of patients with various types of headaches. Telemedicine for headache care has demonstrated high levels of patient satisfaction and is viewed as a viable alternative to in-person care [9]. Multiple randomized controlled studies have shown no difference in the outcome of treatment for headache or in patient satisfaction when comparing telemedicine and in-person care [10, 16–19]. Although patients in the headache category differed from the others in terms of age, sex, and visit type in our study, they did not demonstrate significantly different acceptability scores.

Movement disorders are another neurologic condition with robust literature supporting the use of TN. There have been multiple TN studies in patients with Parkinson’s disease that have been favorable towards telemedicine. For example, a 2010 study demonstrated an increase in quality of life in patients with Parkinson’s disease after telemedicine when compared to an in-person cohort [12]. Other studies have not found this increase in quality of life but have found telemedicine to be equivalent to in-person care when measured based on rates of satisfaction and patient outcome [20–23]. Telemedicine services have even been successfully initiated for the management of deep brain stimulation (DBS) and may provide a much-needed increase in access to DBS for patients with Parkinson’s disease [24]. Developments in mobile health technologies that allow monitoring of mobility in patients with neurologic disease may further improve clinical care and work in conjunction with telemedicine services such as those utilized in Parkinson’s disease [25]. Our movement disorder group included patients with multiple types of movement disorders and provides further evidence that TN is acceptable to patients with these diagnoses.

The third most common ICD10 category was symptoms. This category was used when the consulting neurologist listed a neurological symptom rather than an official diagnosis. Examples of symptoms in this category include dizziness, vision changes, and weakness. Given that ICD10 codes at the time of the first consult were used, it is possible that some of these patients in the symptoms category would eventually receive a more specific diagnosis. Nonetheless, this category is important as it demonstrates that even patients who did not have a definitive diagnosis at the close of their initial neurology consultation were largely satisfied and found their TN visit to be acceptable. There are many patients who have an apparent neurologic symptom of unknown cause. It is valuable to understand what their experience with TN is and how it compares to patients with a known diagnosis.

This study is not without limitations. We were not able to statistically compare acceptability scores across a wide range of ICD10 groups due to the small sample size in some of the categories. However, as can be seen in supplemental file Table 2, the data demonstrates similar acceptability ratings across the different ICD10 groups which suggests that neurologic diagnosis is not a major driver of how likely patients are to accept TN. The distribution of diagnoses in this sample is similar to that seen in referrals to non-VA neurologists at these facilities (data not shown), suggesting that the patients receiving TN care have similar proportions of diagnosis groups as those being referred for in-person care. Reflecting the Veteran population, there were fewer female Veterans and most patients were White, which to some extent reflects the focus of NTNP on rural areas as well as those with reduced neurology access in general. Although this might limit the generalizability of our study, no differences in acceptability relative to race were found. Another limitation is that this data is based on the patient’s first visit with a neurologist. Thus, the diagnosis they receive and their experience may evolve as further follow-up and diagnostic work-up is obtained. Finally, this study does not reflect the experience of the referring provider with Teleneurology care, although our surveys of primary care providers have shown high levels of satisfaction with the NTNP program (data not shown); this is perhaps not surprising in settings with little access to specialty neurology care and considering that VA Teleneurology care uses the same electronic health record as the referring provider, thus limiting communication difficulties across healthcare systems.

In summary, this study demonstrated that patients with different neurologic conditions found their TN experience quite acceptable and that this acceptability was independent of diagnosis but was related to medical comorbidity as patients with higher comorbidities reported higher acceptance of TN care. This suggests that TN care is acceptable to patients with neurologic conditions and symptoms and may be especially acceptable to medically complex patients. Future studies should further evaluate patient experience with TN care to include follow-up care and to examine differences in management and outcomes compared to in-person care.

### Supplementary Information

Below is the link to the electronic supplementary material.

Supplementary file1 (DOCX 27 KB)

## Data Availability

Since these data were collected for clinical and operational purposes, data sharing outside of the VHA system is not permitted.
