# Supplementary material for: Patient acceptance of teleneurology across neurologic conditions
Source: J Neurol. 2024 Feb 23;271(5):2850–8. doi: 10.1007/s00415-024-12200-y (PMC11055742; doi:10.1007/s00415-024-12200-y)
Supplement: Supplementary file 1 — Supplementary file1 (DOCX 27 KB) [file 415_2024_12200_MOESM1_ESM.docx]

Supplemental Files

**Table 1**: Questions included in the patient survey.

| **Patient experience and attitudes toward telehealth care:** | | | | | | | | | | | |
| --- | --- | --- | --- | --- | --- | --- | --- | --- | --- | --- | --- |
|  | Strongly disagree -1 | | 2 | | 3 | | | 4 | | Strongly agree - 5 | |
| I was able to see the provider clearly by video. |  | |  | |  | | |  | |  | |
| I was able to hear the provider clearly by video. |  | |  | |  | | |  | |  | |
| I was able to ask questions directed to the Neurologist. |  | |  | |  | | |  | |  | |
| My provider explained things to me in a way that was easy to understand. |  | |  | |  | | |  | |  | |
| My provider listened to me during the appointment in a caring manner. |  | |  | |  | | |  | |  | |
| In general, telehealth reduces the need to travel long distances in order to meet with my provider |  | |  | |  | | |  | |  | |
| In general, video visits help me get care that I couldn’t access otherwise |  | |  | |  | | |  | |  | |
| **Overall patient satisfaction and acceptability:** | | | | | | | | | | | |
|  | Not at all-1 | 2 | | 3 | | 4 | 5 | | 6 | | Extremely-7 |
| Overall, how satisfied were you with your Teleneurology visit? |  |  | |  | |  |  | |  | |  |
| To what extent was this Teleneurology consult meeting like a face-to-face meeting? |  |  | |  | |  |  | |  | |  |
|  | Definitely not-1 |  | |  | |  |  | |  | | Definitely-7 |
| Would you recommend Teleneurology to other Veterans like yourself? |  |  | |  | |  |  | |  | |  |

**Table 2:** Descriptive data of the three primary acceptability questions by additional ICD10 categories.

|  | Abnormal Scan | Dementia | Multiple Sclerosis | Neuromuscular Disease | Neuropathy/ Radiculopathy | Other | Pain | Seizures/ Epilepsy | Sleep Disorder | Stroke/ TIA | Traumatic Brain Injury | Tumors |
| --- | --- | --- | --- | --- | --- | --- | --- | --- | --- | --- | --- | --- |
| **Overall, how satisfied were you** **with your Teleneurology visit?** |  |  |  |  |  |  |  |  |  |  |  |  |
| N | 4 | 15 | 5 | 1 | 27 | 21 | 9 | 18 | 5 | 18 | 3 | 2 |
| Mean (SD) | 6.5 (1.0) | 6.8 (0.4) | 6.0 (0.7) | 7.0 (n/a) | 5.9 (1.5) | 6.7 (0.5) | 6.2 (1.3) | 6.2 (1.0) | 6.2 (0.8) | 6.1 (1.2) | 6.0 (1.0) | 7.0 (0) |
| **To what extent was this Teleneurology consult like a face-to-face meeting?** |  |  |  |  |  |  |  |  |  |  |  |  |
| N | 4 | 14 | 5 | 1 | 23 | 19 | 9 | 17 | 5 | 16 | 3 | 1 |
| Mean (SD) | 6.3 (0.5) | 6.1 (1.0) | 5.6 (0.9) | 5.0 (n/a) | 5.3 (1.8) | 6.2 (1.1) | 4.9 (1.5) | 5.7 (1.4) | 6.4 (0.9) | 6.1 (1.2) | 5.3 (1.2) | 6.0 (n/a) |
| **Would you recommend Teleneurology to other Veterans like yourself?** |  |  |  |  |  |  |  |  |  |  |  |  |
| N | 4 | 14 | 5 | 1 | 26 | 20 | 9 | 18 | 5 | 17 | 3 | 1 |
| Mean (SD) | 5.5 (2.4) | 6.9 (0.5) | 7.0 (0) | 7.0 (n/a) | 6.0 (1.5) | 6.5 (0.9) | 6.1 (1.2) | 6.4 (1.1) | 6.6 (0.9) | 6.5 (1.3) | 7.0 (0) | 7.0 (n/a) |

**STROBE Statement**: Checklist of items that should be included in reports of cohort studies

|  | **Item No.** | **Recommendation** | **Location** |
| --- | --- | --- | --- |
| **Title and abstract** | 1 | (*a*) Indicate the study’s design with a commonly used term in the title or the abstract | Abstract Methods |
|  |  | (*b*) Provide in the abstract an informative and balanced summary of what was done and what was found | Abstract Methods and Results |
| Introduction | | |  |
| Background/rationale | 2 | Explain the scientific background and rationale for the investigation being reported | Introduction Paragraph Three |
| Objectives | 3 | State specific objectives, including any prespecified hypotheses | Introduction Paragraph Three |
| Methods | | |  |
| Study Design | 4 | Present key elements of study design early in the paper | Methods under VA National Teleneurology Program, Patient Selection, and Patient Satisfaction Survey |
| Setting | 5 | Describe the setting, locations, and relevant dates, including periods of recruitment, exposure, follow-up, and data collection | Methods under VA National Teleneurology Program andPatient Selection |
| Participants | 6 | (*a*) Give the eligibility criteria, and the sources and methods of selection of participants. Describe methods of follow-up | Methods under Patient Selection |
|  |  | (*b*) For matched studies, give matching criteria and number of exposed and unexposed | N/A |
| Variables | 7 | Clearly define all outcomes, exposures, predictors, potential confounders, and effect modifiers. Give diagnostic criteria, if applicable | Methods under Outcomes and Covariates |
| Data Sources/Measurement | 8 | For each variable of interest, give sources of data and details of methods of assessment (measurement). Describe comparability of assessment methods if there is more than one group | Methods under Outcomes and Covariates |
| Bias | 9 | Describe any efforts to address potential sources of bias | Methods under Statistical Analysis |
| Study size | 10 | Explain how the study size was arrived at | Methods under Patient Selection |
| Quantitative Variables | 11 | Explain how quantitative variables were handled in the analyses. If applicable, describe which groupings were chosen and why | Methods under Outcomes and Covariates |
| Statistical Methods | 12 | (*a*) Describe all statistical methods, including those used to control for confounding | Methods under Statistical Analysis |
|  |  | (*b*) Describe any methods used to examine subgroups and interactions | Methods under Statistical Analysis |
|  |  | (*c*) Explain how missing data were addressed | Methods under Statistical Analysis |
|  |  | (*d*) If applicable, explain how loss to follow-up was addressed | N/A |
|  |  | (*e*) Describe any sensitivity analyses | Methods under Statistical Analysis |
| Results | | |  |
| Participants | 13* | (a) Report numbers of individuals at each stage of study—eg numbers potentially eligible, examined for eligibility, confirmed eligible, included in the study, completing follow-up, and analysed | Results Paragraph One |
|  |  | (b) Give reasons for non-participation at each stage | Results Paragraph One |
|  |  | (c) Consider use of a flow diagram | Figure One |
| Descriptive Data | 14* | (a) Give characteristics of study participants (eg demographic, clinical, social) and information on exposures and potential confounders | Results Paragraph Two and Table One |
|  |  | (b) Indicate number of participants with missing data for each variable of interest | N/A |
|  |  | (c) Summarise follow-up time (eg, average and total amount) | N/A |
| Outcome Data | 15* | Report numbers of outcome events or summary measures over time | Results Paragraph Three |
| Main results | 16 | (*a*) Give unadjusted estimates and, if applicable, confounder-adjusted estimates and their precision (eg, 95% confidence interval). Make clear which confounders were adjusted for and why they were included | Results Paragraph Three |
|  |  | (*b*) Report category boundaries when continuous variables were categorized | Results Paragraph Three |
|  |  | (*c*) If relevant, consider translating estimates of relative risk into absolute risk for a meaningful time period | N/A |
| Other analyses | 17 | Report other analyses done—eg analyses of subgroups and interactions, and sensitivity analyses | Results Paragraph Three |
| Discussion | | |  |
| Key results | 18 | Summarise key results with reference to study objectives | Discussion Paragraph One |
| Limitations | 19 | Discuss limitations of the study, taking into account sources of potential bias or imprecision. Discuss both direction and magnitude of any potential bias | Discussion Paragraph Six |
| Interpretation | 20 | Give a cautious overall interpretation of results considering objectives, limitations, multiplicity of analyses, results from similar studies, and other relevant evidence | Discussion Paragraph Seven |
| Generalisability | 21 | Discuss the generalisability (external validity) of the study results | Discussion Paragraph Seven |
| Other Information | | |  |
| Funding | 22 | Give the source of funding and the role of the funders for the present study and, if applicable, for the original study on which the present article is based | Declarations |
